# Bioactive and Functional Properties of *Ferulago angulata* Essential Oil for Sustainable Food Packaging Systems

**DOI:** 10.1002/fsn3.72470

**Published:** 2026-10-19

**Authors:** Mahshid Bahraminejad, Delara Moradi Mirhesari, Ehsan Sadeghi, Behnam Bahramian, Reza Abedi‐Firoozjah, Samira Shokri

**Affiliations:** ^1^ Student Research Committee Kermanshah University of Medical Sciences Kermanshah Iran; ^2^ Department of Food Science and Technology, School of Nutrition Sciences and Food Technology, Research Center for Environmental Determinants of Health (RCEDH), Health Institute Kermanshah University of Medical Sciences Kermanshah Iran; ^3^ Department of Food Sciences and Technology, Faculty of Nutrition and Food Sciences Tabriz University of Medical Sciences Tabriz Iran; ^4^ Nutritional Health Research Center Lorestan University of Medical Sciences Khorramabad Iran

**Keywords:** bioactive agent, controlled release, encapsulation, essential oil, *Ferulago angulata*, nanoemulsification

## Abstract

*Ferulago angulata* essential oil (FAEO) has attracted increasing attention as a natural bioactive agent for sustainable food packaging applications due to its antimicrobial and antioxidant properties. FAEO contains several functional phytochemicals, including α‐pinene (21.85%–35.08%), limonene (4.8%–21.85%), sabinene (16.9%), and (Z)‐β‐ocimene (18.0%–35.5%), which contribute to its antioxidant and antimicrobial activities against foodborne pathogens such as *Bacillus thuringiensis* , *Escherichia coli* O157:H7, and *Staphylococcus aureus* . Reported minimum inhibitory concentration (MIC) values range from 0.45 to 10 mg/mL, while antioxidant activity includes 22% DPPH radical scavenging and 52.81% ABTS radical inhibition. FAEO has been incorporated into a wide range of polymeric materials, such as sodium alginate, chitosan, gelatin, zein, potato starch, and cellulose‐based films. Advanced encapsulation approaches, including nanoemulsification, electrospinning, and freeze‐drying, have improved the stability, controlled release, and bioactivity of FAEO in packaging systems. Here, we review FAEO's role in active packaging systems, detailing how it affects film properties such as structural, physical, mechanical, and barrier properties. Further, FAEO‐loaded systems demonstrate controlled release driven by polymer type, morphology, and encapsulation method, thereby influencing their antimicrobial and antioxidant performance. Unlike phenolic‐rich essential oils (EO_s_ such as thyme and oregano, which exhibit strong antioxidant potential but intense sensory profiles), FAEO offers a milder aroma and different antimicrobial mechanisms, potentially suitable for packaging applications where sensory neutrality is desirable. Despite these advantages, several challenges remain, including volatility, hydrophobicity, sensory impacts, regulatory limitations, and scalability for industrial applications. Overall, FAEO shows promise as a natural alternative to synthetic preservatives for the development of sustainable and active food packaging systems. However, future studies should address industrial scalability, real‐time shelf‐life evaluations, and the optimization of FAEO‐incorporated biodegradable films.

## Graphical abstract

*Ferulago angulata* essential oil (FAEO) contains monoterpenes such as α‐pinene, limonene, sabinene, and (Z)‐β‐ocimene, which contribute to its antimicrobial activity and moderate antioxidant properties. Incorporation of FAEO into biopolymer‐based films and encapsulation systems can improve its stability, dispersion, and controlled release. Techniques such as nanoemulsification, electrospinning, spray drying, and nanoparticle encapsulation have been investigated to enhance the stability, bioactivity, and release behavior of FAEO in food packaging systems. These FAEO‐loaded films and coatings can help extend food shelf life by inhibiting microbial growth and reducing oxidative deterioration. However, FAEO has several limitations, including high volatility, hydrophobicity, and poor water solubility, potential sensory effects on food, regulatory challenges, and difficulties associated with industrial‐scale production and standardization. Therefore, optimization of encapsulation techniques, polymer matrices, and processing conditions is necessary to achieve stable FAEO incorporation and controlled release in practical food packaging applications. The graphical abstract was created with the assistance of ChatGPT and has been reviewed and approved by all authors.

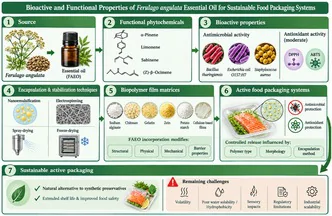

## 1 Introduction

Over the past two decades, the international food sector has undergone a significant shift towards packaging and food products that are not only more sustainable but also safe and long‐lasting. This transformation is driven by the increasing demand for biodegradable and environmentally friendly packaging materials to replace petroleum‐based materials, which are typically non‐biodegradable and associated with environmental and health concerns (Tian et al. 2023). Consequently, the demand for natural, biodegradable, and non‐toxic alternatives has increased as they offer enhanced food safety and quality and the prospect of reducing environmental consequences. One of the potential sources to improving these issues in food bio‐preservation is the introduction of EO_s_, such as those derived from thyme and oregano into food packaging systems (Bibow and Oleszek 2024). EOs have been found to have bioactive properties; in particular, antimicrobial activity and antioxidant activity; hence, they can act as a natural additive when they are used as additives in the active food packaging (Cesca et al. 2024; Shokri et al. 2020).

Active packaging has been gaining increasing attention in recent years. It is the next step in packaging technology where the packaging is constructed of materials that can interact with the food inside or with the immediate environment. One way to obtain an active system is to embed EOs in packaging materials. Such a system could facilitate food preservation by not only forming a physical barrier but also by inhibiting the growth of microorganisms and the oxidative spoilage of food (Bibow and Oleszek 2024; Esmaeili and Ebrahimzadeh Fazel 2016). Numerous studies have been conducted in order to support the use of EOs in food preservation. It has been demonstrated that EOs are able to extend the lifetime of a variety of food products by not only limiting the growth of spoilage and pathogenic microorganisms but also by the delay of lipid oxidation, which is the main cause of food quality deterioration (Bibow and Oleszek 2024). One of the reasons for these positive effects is the multidimensional character of EOs which mainly consist of terpenes, phenols, and various other aromatic compounds that possess notable biological activities (Cesca et al. 2024). For instance, methylcellulose‐based films containing nanoencapsulated FAEO demonstrated sustained release of the active compounds and antioxidant activity, making them suitable for active food packaging applications (Esmaeili and Ebrahimzadeh Fazel 2016). Additionally, chitosan‐based edible coatings incorporating FAEO nanoemulsion effectively extended the shelf life of rainbow trout fillets up to 16 days by inhibiting microbial growth and reducing lipid peroxidation (Shokri et al. 2020). These confirm that EO‐incorporated packaging systems can actively preserve food quality and extend product shelf life.

Unfortunately, thymol's performance in packaging systems is still a matter of volatility and interactions with the packaging matrix and food components of their chemical structure. Sivaram et al. (2022) noted that thymol's practical use is restricted by its pungent smell, poor aqueous solubility, and susceptibility to air, light, heat, along with its rapid evaporation rate (Sivaram et al. 2022).

The enormous potential of EO, however, is hampered by the practical application in food packaging with issues such as stability and controlled release of EO, the possibility of changing food sensory properties, and the issue of achieving homogeneous distribution in packaging materials (Bibow and Oleszek 2024). New methods of stabilization and improvement in the performance of EO‐containing food packaging are new materials science advances, such as the creation of biopolymer‐based films and the nano‐encapsulation technique (Cesca et al. 2024; Razmjoo et al. 2022). FAEO is a component of traditional medicine and is characterized by a diverse profile of monoterpenes and sesquiterpenes, which contribute to its bioactivity. These characteristics highlight the potential of FAEO for use in food packaging, particularly in active packaging systems aimed at extending shelf life and ensuring food safety (Razmjoo et al. 2022; Shahbazi et al. 2016).

The application of essential oils (EOs) in packaging materials is in line with the general trend of sustainable and functional packaging. Biopolymer‐based films are the main carriers of EOs, and one can consider films made of chitosan as the carriers of EOs, which are being tested increasingly (Thiyagamoorthy et al. 2024).

Nonetheless, the effective use of FAEO in packaging material systems is dependent on an in‐depth knowledge of its constituent chemistry, its functional characteristics, and how the EO interact with the packaging material (Shokri et al. 2020). While EOs from plants such as cinnamon, clove, thyme, and oregano have been extensively studied for food packaging applications (Cesca et al. 2024), the potential of FAEO remains largely unexplored. Unlike commonly studied EO that are rich in phenolic compounds (e.g., thymol, eugenol, carvacrol), FAEO is characterized by high levels of monoterpene hydrocarbons such as α‐pinene, sabinene, and (Z)‐β‐ocimene (Sefidkon and Omidbaigi 2004). This unique chemical composition offers distinct advantages. However, the lower phenolic content also results in moderate antioxidant activity compared to phenolic‐rich oils (Ghasemi Pirbalouti et al. 2016). Existing research on clove essential oil (CEO) for active packaging is scattered, especially concerning polymer selection, encapsulation methods, dosages, and efficacy measures. Incorporating CEO into polymer matrices boosts functional and technical properties while supporting sustainable packaging objectives. CEO‐based packaging reduces food spoilage and chemical use, supporting SDG 12 and 13. Advanced systems like nanoencapsulation allow precise bioactive release, aligning with Industry 4.0–6.0 smart manufacturing (Godini et al. 2026). These strategies promote sustainability and bridge research with scalable solutions. This review provides an integrated structure–function analysis that directly links polymer matrix characteristics, release mechanisms, and shelf‐life performance of FAEO‐loaded packaging systems across diverse food systems.

Therefore, this review aims to address the current literature gap by providing an extensive discussion of FAEO for food packaging applications, including its chemical profile, functional properties, incorporation techniques (encapsulation, nanoemulsification, electrospinning), effects on packaging material properties, and existing applications, challenges, and future perspectives. By utilizing the latest scientific research and highlighting both the potential and limitations of FAEO‐loaded packaging materials, this review seeks to contribute to the development of safe, sustainable, and innovative food packaging solutions that meet the demands of contemporary consumers and the food industry.

## 2 Literature Search Strategy

This review was conducted as a narrative review of the available literature on FAEO and its applications in food packaging systems. A comprehensive literature search was performed using the following electronic databases: PubMed, Scopus, Web of Science, and Google Scholar. The search was conducted between January 2000 and 2026. Keywords and their combinations included: (“*Ferulago angulata*”) OR (“Chavil”) OR (“essential oil”) OR (“Ferulago angulate essential oil”) OR (“chemical composition”) OR (“Active packaging”) OR (“edible coating”) OR (“packaging films”) OR (“antioxidant activity”) OR (“antimicrobial activity”) OR (“encapsulation”) OR (“nanoemulsion”) OR (“electrospinning”) OR (“edible film”) OR (“food preservation”) AND (“meat”) OR (“seafood”) OR (“Turkey”) OR (“butter”).

Four researchers initially screened articles by examining titles and abstracts, then assessed full texts. The corresponding authors validated the inclusion/exclusion criteria and data extraction methods. Only original English research directly examining FAEO in active packaging systems was included. Finally, endNote software was used to manage and screen the titles and abstracts obtained from databases.

Inclusion criteria were: (i) peer‐reviewed original research articles; (ii) studies reporting on FAEO's chemical composition, bioactivity, encapsulation, or food packaging applications; (iii) articles published in English; (iv) published between 2000 and 2026; and (v) full‐text articles available.

Exclusion criteria were: (i) review articles, systematic reviews, meta‐analyses, conference abstracts, editorials, and book chapters; (ii) studies not directly related to *F. angulata* or food packaging; (iii) articles published in languages other than English.

This review article does not involve any human or animal subjects. All data were obtained from previously published peer‐reviewed literature. Therefore, no ethical approval was required for this study.

## 3 Chemical Composition of FAEO

Composition and amount of compounds can vary from part of the plant (Reidel et al. 2017). Bagherifar et al. (2019) analyzed nine *F. angulata* populations from different regions of Iran and reported that α‐pinene content varied significantly from 20.84% to 49.06% depending on geographical location and altitude (Bagherifar et al. 2019). This variation is attributed to differences in soil properties and climatic conditions (temperature, precipitation) across regions. Rustaiyan et al. (2002) analyzed the essential oil of *Ferulago angulata* aerial parts and identified 25 components, with β‐phellandrene (32.0%) and α‐phellandrene (13.8%) as the major constituents (Rustaiyan et al. 2002). A study of the FAEO stem oil reported α‐pinene as the main component (Akhlaghi 2008).

Further, another study showed that FAEO contains the following compounds (Figure 1 and Table 1). The chemical composition of FAEO has been extensively studied. The commonly reported compounds identified by GC–MS analysis include α‐pinene, (Z)‐β‐ocimene, sabinene, β‐phellandrene, α‐phellandrene, and limonene, with their relative percentages varying significantly across different studies (Akhlaghi 2008; Rustaiyan et al. 2002; Sefidkon and Omidbaigi 2004).

**FIGURE 1:**
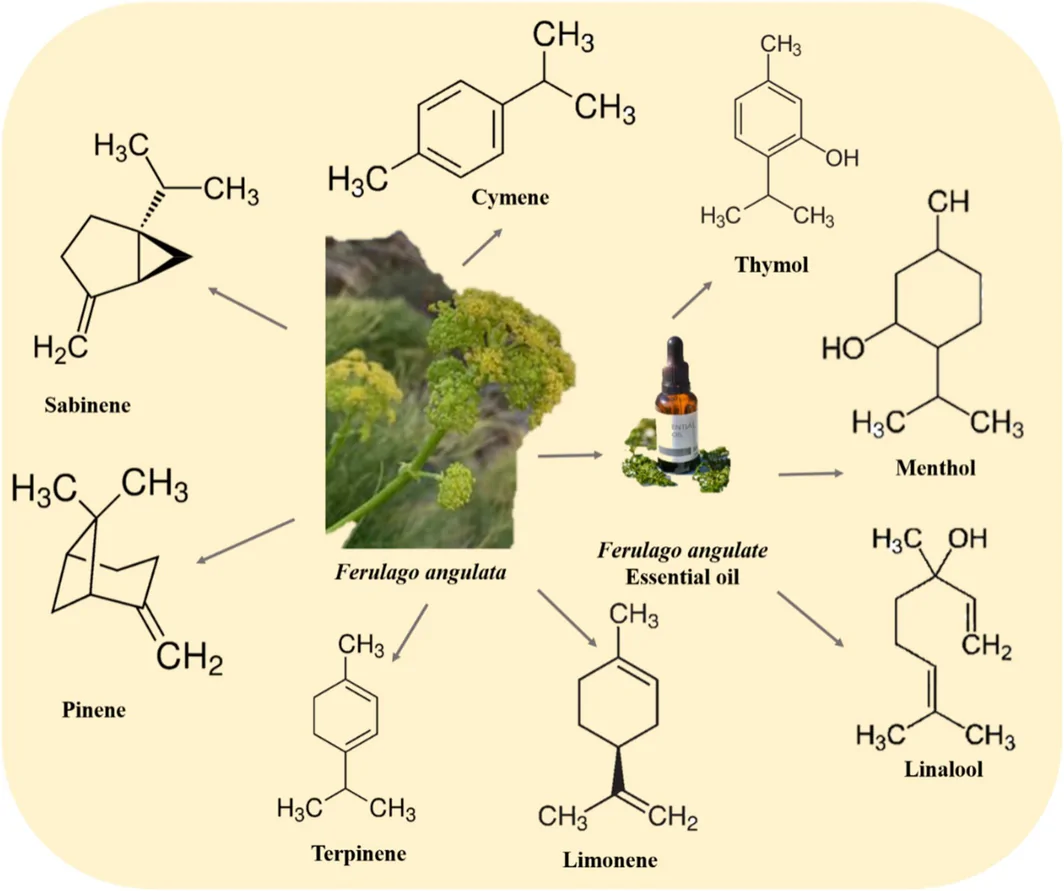
Chemical structures of representative monoterpenes identified in FAEO (Akhlaghi 2008; Rustaiyan et al. 2002; Sefidkon and Omidbaigi 2004).

**TABLE 1:** Major chemical components of FAEO reported in different studies.

Component	Sefidkon and Omidbaigi (2004)	Akhlaghi (2008)	Rustaiyan et al. (2002)
α‐Pinene	24.2%	12.24%–21.20%	9.1%
(Z)‐β‐Ocimene	18.0%	2.49%–6.04%	—
Sabinene	16.9%	0.50%–2.37%	2.1%
β‐Phellandrene	0.1%	15.79%–16.62%	32.0%
α‐Phellandrene	2.5%	18.14%–27.24%	13.8%
Limonene	4.8%	—	—

The effect of plant part on chemical composition has been demonstrated by Narimani et al. (2022), who reported that flower oils of three Ferulago species (including *F. angulata* ) were dominated by (Z)‐β‐ocimene (38.46%–47.21%) and α‐pinene (10.25%–18.32%); leaf oils contained α‐pinene (15.01%–22.16%), β‐phellandrene (2.48%–14.73%), and α‐phellandrene (0.52%–13.8%), while fruit oils exhibited the highest compositional diversity, with (Z)‐β‐ocimene (10.21%–41.19%), α‐phellandrene (7.51%–31.89%), and α‐pinene (8.96%–17.71%) as major components. Essential oil yield also varied by plant part, ranging from 0.46% to 2.65% (v/w) (Narimani et al. 2022).

Several predominant compounds found in FAEO exhibit well‐documented bioactivities. For instance, α‐pinene has shown inhibitory activity against *Listeria monocytogenes* (Ghasemi Pirbalouti et al. 2016; Taran et al. 2010) and against *Staphylococcus aureus* (Taran et al. 2010).

Impact of compositional differences on functional performance has been established. The ‘Mongar’ population had the highest α‐pinene content (49.06%) and also exhibited the strongest antiradical activity (EC_50_ = 42.07 μg/mL), which was attributed to its high total phenolic and flavonoid contents (Bagherifar et al. 2019). These findings underscore that standardization of plant source, region, plant part, and extraction protocol is essential for consistent functional performance in food packaging applications. In conclusion, FAEO is characterized by a diverse chemical composition whose biological activities justify its use in food preservation systems and may have beneficial effects for consumers (Table 1).

## 4 Functional Properties of FAEO

The functional characteristics of FAEO are most aligned with their future use in food packaging systems, where they may be utilized as natural additives and active preservatives. The following section gives a broad overview of FAEO functional characteristics, including antioxidant, antimicrobial, and organoleptic effects, and effects on food matrix characteristics.

### 4.1 Antioxidant Potential

Among the most notable of FAEO functional attributes is their antioxidant activity. The antioxidant capacity is mostly owed to the rich presence of phenolic compounds, which are highly effective free radical scavengers and prevent oxidizing reactions. In studies using the ABTS radical scavenging assay, encapsulated FAEO showed considerable antioxidant capacity, with maximum free radical scavenging in formulations of 3% EO encapsulated using maltodextrin and Arabic gum, achieving up to 52.81% inhibition. Their total phenolic contents, measured to determine antioxidant activity, were highest in these formulations, at a value of 66.88 mg/mL (Salehi et al. 2021).

Essential oils rich in phenolic compounds, such as thyme and oregano, are generally known for their antioxidant activity (Bibow and Oleszek 2024; Tian et al. 2023). The moderate activity of FAEO is attributed to its high monoterpene hydrocarbon content (α‐pinene, limonene, cis‐β‐ocimene) and low phenolic compound levels, whereas phenolic‐rich oils such as thyme and oregano contain thymol and carvacrol which are more potent radical scavengers (Ghasemi Pirbalouti et al. 2016). This significant difference is attributed to FAEO's low phenolic content and dominance of monoterpene hydrocarbons (α‐pinene, cis‐β‐ocimene), which are weak radical scavengers compared to phenolic compounds such as thymol and carvacrol (Ghasemi Pirbalouti et al. 2016). Even the thymol‐rich Kallar chemotype of FAEO (containing 6.9% thymol) showed an IC₅₀ of 488 μg/mL (Ghasemi Pirbalouti et al. 2016). In another study, chitosan coating solutions containing 3% FAEO showed a DPPH radical scavenging activity of 22%, compared to 6.15% for chitosan alone (Shokri et al. 2020). Nanoemulsification of FAEO significantly boosted antioxidant activity due to better dispersion and increased surface area, enhancing free radical scavenging efficiency (Shokri et al. 2020).

### 4.2 Antimicrobial Effects

The antimicrobial activity of FAEO is another important functional attribute, more specifically for application in food preservation and safety. GC–MS studies have revealed a mixture of compounds with bioactivity in FAEO, including (Z)‐β‐ocimene, α‐pinene, p‐cymene, sabinene, and some phellandrenes (Farokhi‐Firoozi et al. 2020; Moghaddam et al. 2018).

For instance, FAEO demonstrated antibacterial activity against *Bacillus thuringiensis* with a minimum inhibitory concentration (MIC) of 8 μL/mL (Moghaddam et al. 2018). Against foodborne pathogens, FAEO showed MIC values of 5 mg/mL for *E. coli* O157:H7 and 10 mg/mL for *S. aureus* (Panahi and Mohsenzadeh 2022). This difference is attributed to the structural characteristics of the bacterial cell envelope: Gram‐positive bacteria possess a thicker peptidoglycan layer but lack the outer membrane, allowing easier penetration of hydrophobic monoterpenes. In contrast, Gram‐negative bacteria have an additional outer membrane composed of lipopolysaccharides (LPS) that acts as a permeability barrier, limiting the entry of hydrophobic compounds into the cell (Nazzaro et al. 2013). Further, FAEO exhibited antifungal effects, completely inhibiting the growth of Fusarium oxysporum at the highest concentration tested (Moghaddam et al. 2018). In fish preservation, chitosan coatings containing FAEO significantly reduced the counts of *Shewanella putrefaciens* and *Pseudomonas fluorescens* , two major spoilage bacteria (Shokri et al. 2020).

These compounds are reported to possess promising antibacterial and antifungal activities against a number of pathogens and spoilage microbes. For example, the EO inhibited bacteria including *B. thuringiensis* (MIC value of 8 μL/mL) and presented activity against some phytopathogenic fungi, including *F. oxysporum*, which showed 100% inhibition at the highest concentration (Moghaddam et al. 2018). The mechanism of the antimicrobial activity is presumed to be through disruption of microbial cell membranes, interference with metabolism, and inhibition of spore germination, largely as a consequence of its monoterpene and phenolic compounds having a synergistic action. The antimicrobial mechanism of FAEO involves multiple pathways beyond simple membrane disruption. (i) Monoterpenes insert into the phospholipid bilayer, increasing membrane fluidity and permeability, leading to leakage of intracellular contents and cell death (Burt 2004), as confirmed by scanning electron microscopy imaging showing membrane destruction (Hamidian et al. 2024). (ii) FAEO induces oxidative stress by generating reactive oxygen species (ROS) including superoxide anion ($O_{2}^{-}$) and hydrogen peroxide (H_2_O_2_), which damage bacterial DNA, proteins, and membrane lipids (Bowbe et al. 2023). (iii) Furthermore, ethanol extracts of *F. angulata* have demonstrated antifungal activity against *Candida albicans* (Alizadeh et al. 2017). In applied food sciences, the use of FAEO in food items or packaging materials has been found to minimize microbial load and improve the shelf life of products. For instance, incorporation of FAEO into probiotic yogurt not only enhanced the viability of health‐promoting bacteria during storage but also contributed to the microbial safety of the final product (Keshavarzi et al. 2021).

### 4.3 Sensory and Organoleptic Effects

Aside from its preservational activities, FAEO has the ability to affect the sense characteristics of food systems. The EO contributes a defining flavor and aroma profile, which tends to improve the organoleptic attributes of some foods. Use of FAEO at low concentrations (0.01%) in studies on probiotic yogurt enhanced the sensory attributes of taste, aroma, and acceptability when compared with controls (Keshavarzi et al. 2021). At greater concentrations, it has the potential to overpower flavors or develop bitterness, and thus requires optimization for every application. The volatile compounds of FAEO, including (Z)‐β‐ocimene and α‐pinene, are accountable for its aroma, which is able to cover up unpleasant odors in pre‐packaged food. Such an application is particularly beneficial in packaging, in which the EO will play a part in both the preservation and the attraction of the product (Keshavarzi et al. 2021).

### 4.4 Neuroprotective and Health‐Promoting Properties

While the primary focus of this review is food packaging, it is worth noting that FAEO has demonstrated additional health‐promoting properties in pharmacological studies. For instance, inhalation of FAEO has been shown to exhibit anxiolytic and antidepressant‐like effects in animal models, potentially due to interactions of its monoterpene components with GABA receptors (Bagci et al. 2016). Additionally, the antioxidant activity of *Ferulago angulata* extracts may contribute to reducing oxidative stress (Rafieian‐Kopaei et al. 2014). However, these properties are not directly relevant to food packaging applications and are mentioned only briefly to highlight the broader bioactivity profile of FAEO. Further details are beyond the scope of this review.

Though contemporary research is required for confirmation, traditional use for digestive disorders points to spasmolytic or anti‐ulcer effects (Table 2) (Rahimpour et al. 2021).

**TABLE 2:** Functional properties of FAEO.

Functional property	Key attributes	Mechanism of action	Application
Antioxidant activity	Moderate activity antioxidant capacity due to coumarins, flavonoids, and monoterpenes	Free radical scavenging, inhibition of lipid peroxidation	Natural antioxidants in packaging, food fortification
Antimicrobial activity	Strong antibacterial and antifungal properties	Disrupts microbial membranes, inhibits germ tube formation, affects ergosterol biosynthesis and mitochondrial function	Food preservation and safety
Sensory and Organoleptic Effects	Flavor Profile	Aromatic compounds (monoterpenoids and sesquiterpenoids), enhances taste and aroma, improves acceptability, olfactory stimulation	Food flavoring and food production
Neuroprotective and Health‐Promoting Properties	Sedative effects, neuroprotection, potential for Alzheimer's disease	Acetylcholinesterase (AChE) inhibition, antioxidant activity, reduction of oxidative stress	Health‐functional foods
Synergistic Effects and Mechanisms	Chemical Interactions, diverse bioactivities from various compounds (73 identified molecules)	Free radical scavenging, membrane modification, enzyme inhibition	Active functional products

### 4.5 Synergistic Effects and Mechanisms of Action

The significant bioactivities of monoterpenes, sesquiterpenes, and coumarins include free radical scavenging and enzyme inhibition (Farokhi‐Firoozi et al. 2020). These compounds have synergistic effects that provide functional benefits that make FAEO a resourceful component for food preservation. The functions of FAEO are complex and relevant for food preservation. Their antimicrobial activity, along with their moderate antioxidant properties and effects on sensory attributes and matrix stability in food, make them potential natural alternatives for synthetic preservatives. FAEO also enhances probiotic viability and improves the health benefits of functional food products (Keshavarzi et al. 2021). Further, advanced formulations have led to improvements in both stability and efficacy of FAEO, which may enhance potential application in functional food preservation.

## 5 Methods for Obtaining Films Loaded With FAEO

FAEO has been traditionally used for a long time by adding to dairy products to prevent them from deterioration. The direct application of EO in food presents challenges despite their biological properties. High concentrations of EO in food can lead to undesirable organoleptic properties (Ghasemi Pirbalouti et al. 2016; Keshavarzi et al. 2021). For this purpose, various techniques have been investigated for the incorporation of EO as a practical approach to improve their application, stability, and efficacy. These techniques include nanoencapsulation, microencapsulation, electrospinning, and incorporation into food packaging and coatings.

To improve the application of active compounds in FAEO or protect them from adverse environmental conditions, encapsulation has been considered an achievable approach. encapsulation is defined as a carrier matrix that entraps a bioactive material, allowing control over the rate of its release (Shishir et al. 2018). These methods are frequently used for encapsulating both hydrophilic and hydrophobic compounds; however, they provide limited control over the particle size and structure of the emulsion and are useful only to a restricted range of matrices (Joye and McClements 2014). Although several approaches for encapsulating biologically active compounds have been proposed in the literature, these approaches cannot be deemed standard or suitable for the encapsulation of all biologically active compounds.

### 5.1 Electrospinning Method

Electrospinning and electrospraying methods are effective for the encapsulation of active components, and they offer the added advantage of low‐temperature operation (Jia et al. 2016). These electrohydrodynamic techniques will not only allow you to encapsulate active biocomponents such as FAEO, but also to encapsulate these molecules in a way that will limit their exposure to various environmental factors (e.g., pH, light, humidity, oxygen, and enzymes) that may contribute to their degradation. It is still possible to increase the bioavailability of these compounds, mask the taste and/or aroma of these compounds, and provide controlled release of the compounds in the electrospun or electrosprayed systems. Consequently, an important problem is the selection of a suitable polymer for the encapsulation of EO to produce materials that enhance and maintain their bioactive qualities (Coelho et al. 2021). For instance, Shavisi (2024) fabricated zein nanofibers containing FAEO (0.5%–1.5%) via electrospinning. The 1% and 1.5% FEO nanofibers showed better oxidative stability than BHT (100 mg/Kg). The zein‐FEO nanofibers significantly delayed lipid oxidation in butter oil, lowering peroxide, TBARS, p‐anisidine, and acid values, thereby extending oxidative stability during 24 days of accelerated storage (Shavisi 2024).

### 5.2 Solvent Casting

Another method explored in the literature involves the use of the casting technique. Razmjoo et al. (2022), for instance, utilized the casting method to fabricate CMC/ZG composite films incorporated with plant extracts (Razmjoo et al. 2022). This technique of making edible films typically includes: dissolving biopolymers in solvents, performing heat treatment, casting, and drying. The biopolymer and solvent are mixed in the first phase, and then to assist the biopolymer in dissolving in the solvent (Rhim and Ng 2007). Regardless of these benefits, solvent casting remains largely appropriate for lab‐scale research because of its low production rate and possible drawbacks like mechanical strength limitations.

### 5.3 Coating

Edible coatings can carry bioactive substances, thereby extending the shelf life of perishable foods (Shokri et al. 2020; Yang et al. 2026). In a study, Shokri et al. (2020) prepared a chitosan‐based nanoemulsion coating with FAEO (1%–3%) using ultrasonication (20 kHz, 5 min). Nanoemulsification dramatically reduced droplet size, increased ζ‐potential, and lowered polydispersity index. The 3% EO nanoemulsion significantly inhibited *S. putrefaciens* and *P. fluorescens* , retarded TVB‐N and lipid peroxidation, and preserved texture, color, and overall acceptability of trout fillets during 16 days at 4°C (Shokri et al. 2020).

### 5.4 Nanoparticle Encapsulation

Nanoparticle encapsulation of EO has been studied as a practical way to boost their stability and efficacy, offering benefits such as controlled releas*e* (Kaboudi et al. 2023). Accordingly, in a study by Kaboudi et al. (2023), chitosan nanoparticles were prepared by ionic gelation. Chitosan (1% w/v in acetic acid) was mixed with Tween 80 (1:1.10 ratio). FAEO (0–0.6 g in ethanol) was added at CS; FAEO ratios of 1:0 to 1:1.25. The mixture was homogenized at 10,000 rpm for 15 min, then cross‐linked with 0.4% trisodium polyphosphate (TPP; 1:1 v/v), stirred at 400 rpm for 45 min, centrifuged at 10,000 rpm for 40 min, and freeze‐dried (Kaboudi et al. 2023).

## 6 Effect of FAEO Incorporation on the Packaging Material Properties

The antimicrobial activity of FAEO is attributed to its active monoterpene components, such as α‐pinene and sabinene (Esmaeili and Ebrahimzadeh Fazel 2016). Shavisi (2024) encapsulated FAEO in zein nanofibers via electrospinning, with main components being α‐pinene (35.08%) and limonene (21.85%). SEM analysis showed smooth, uniform, cylindrical fibers. Nanofiber mats containing 1% and 1.5% FAEO significantly delayed lipid oxidation in butter during 24 days of accelerated storage. At the end, peroxide values ranged from 0.79 to 1.03 meq O_2_/kg, and TBARS values ranged from 0.35 to 0.45 mg MDA/kg, while p‐anisidine and acid values indicated enhanced oxidative stability, demonstrating the effective antioxidant activity of FEO‐loaded nanofibers (Shavisi 2024). Razmjoo et al. (2022) developed biodegradable films combining Zedo gum (ZG), carboxymethyl cellulose (CMC), and Lemon or Ferulago extracts (LVE/FAE). Incorporating 2.5 μL LVE or FAE increased tensile strength (TS) from 6.92 MPa (for the control CMC/ZG 80:20 film) to approximately 10.55–12.50 MPa, while elongation at break (EAB) decreased from 24.21% to 6.10% for the combined extracts. The addition also significantly reduced moisture content and water vapor permeability. The optimal ratio (20:80 ZG/CMC with 2.5 μL LVE/FAE) yielded improved mechanical, moisture, and barrier properties, making it suitable for edible food packaging with antioxidant activity. The improvement in barrier properties was attributed to ester bond formation and denser structure. FTIR analysis confirmed hydrogen bonding interactions, as evidenced by a shift in the OH stretching peak to 3442 cm^−1^(Razmjoo et al. 2022). Incorporation of FAEO (0.5, 1.0, and 1.5% wt) into starch/CMC films significantly affected physical, mechanical, and chemical properties. Increasing FAEO from 0.5% to 1.5% decreased TS (7.50 → 3.37 MPa) due to disruption of the polymer network and reduced adhesion forces. WVP decreased from 1.81 to 1.14 (×10^−7^ g/m.h.Pa) due to the hydrophobic nature of FAEO, which created tortuous paths for water molecules. Moisture absorption decreased from 14.43% to 10.25%, and solubility decreased from 28.72% to 20.88%, both attributed to hydrogen bonding between FAEO and the polymer matrix, reducing available spaces for water (Beigmohammadi et al. 2019). Esmaeili and Ebrahimzadeh Fazel developed methylcellulose (MC)‐based films with nanoencapsulated FAEO, prepared via ultrasonic bath. Incorporating the nanoemulsion decreased film thickness and tensile strength but increased elongation at break and lightness. The films exhibited antioxidant activity and sustained release of EO. Using Response Surface Methodology, optimal conditions were identified to enhance film thickness, stability, and antioxidant activity (Esmaeili and Ebrahimzadeh Fazel 2016). Chitosan alone showed 6.15% radical scavenging, which increased to 7.2%, 14%, and 22% with 1%, 2%, and 3% FAEO, respectively. FAEO had relatively low antioxidant activity, likely due to low phenolic content and high monoterpenes like α‐pinene and limonene (Shokri et al. 2020). Shahbazi et al. found that FAEO exhibited moderate antibacterial activity, especially against *E. faecalis* (MIC 33.3 μL/mL, inhibition zone 4 mm), with activity varying by region, Kurdestan oil was most effective, while Lorestan was weakest. The FAEO showed less potency than tetracycline and no effect on *K. pneumoniae* . Antioxidant testing via DPPH revealed Lorestan oil had the highest radical scavenging activity, but all were weaker than quercetin. The low antioxidant activity is linked to the absence or low levels of phenolic compounds like thymol, and high monoterpene hydrocarbons such as α‐pinene, which have weak antioxidant activity (Shahbazi et al. 2016).

The choice of polymer matrix significantly influences film performance, as demonstrated by Razmjoo et al. (2022), who investigated carboxymethyl cellulose (CMC) and Zedo gum (ZG) composite films incorporated with *Ferulago angulata* extract (FAE). They reported that the incorporation of FAE into ZG/CMC films increased tensile strength (TS) from 6.92 MPa to approximately 10.55–12.50 MPa, which was attributed to ester bond formation between the extract and polymer matrices, resulting in a denser structure with enhanced cross‐linking (Razmjoo et al. 2022). In contrast, Beigmohammadi et al. (2019) reported that incorporating FAEO into starch/CMC films decreased TS from 7.50 to 3.37 MPa, attributed to the disruption of the polymer network and reduction of intermolecular forces. These divergent effects highlight that the polymer matrix dictates whether FAEO/FAE acts as a reinforcing filler or a disruptive plasticizer, underscoring the importance of matrix selection for optimal film performance (Beigmohammadi et al. 2019).

FAEO was successfully nanoencapsulated in chitosan matrix. Antimicrobial testing revealed that FAEO was more effective against *S. aureus* (Gram‐positive) than *E. coli* (Gram‐negative) due to cell wall structure differences. For *S. aureus*, MIC and MBC values were 0.45 and 0.90 mg/mL, with an inhibition zone of 18.8 mm. For *E. coli* , MIC and MBC were 2.12 and 4.25 mg/mL, with a 10.3 mm zone (Kaboudi et al. 2023). The antibacterial activity of FAEO against *Enterococcus faecalis* showed an inhibition zone of 4 mm and an MIC of 33.3 μL/mL, though it was less potent than tetracycline (Shahbazi et al. 2016). Moghaddam et al. (2018) suggested that the antimicrobial activity of FAEO is related to its monoterpene hydrocarbons, including (Z)‐β‐ocimene (19.93%), α‐pinene (15.50%), and p‐cymene (7.67%), which may interact with the bacterial cell membrane due to their lipophilic nature. However, the exact mechanism of action was not fully elucidated in their study, as they acknowledged that the complex and variable chemical composition of essential oils prevents a clear understanding of the mechanism of action (Moghaddam et al. 2018).

Shokri et al. (2020) evaluated the antimicrobial activity of chitosan and FAEO against fish spoilage bacteria, *Shewanella putrefaciens* and *Pseudomonas fluorescens*. Chitosan alone showed MIC and MBC values at 20% and 40% concentrations. Adding EO at 1% had little effect, but higher concentrations (2%–3%) significantly reduced MIC and MBC, with MBC for Shewanella at 25% and for Pseudomonas at 30% (Shokri et al. 2020). The mechanisms include membrane disruption and nutrient chelation. Nanoemulsification enhanced EO activity, with smaller droplets promoting faster bacterial membrane penetration. Overall, EO exhibited moderate antibacterial activity, improved by nanoemulsification, depending on formulation and concentration (Shokri et al. 2020). Panahi and Mohsenzadeh (2022) also evaluated the antimicrobial activity of sodium alginate edible coating containing FAEO against bacteria that was notable, with MICs between 5 and 40 mg/mL, most effective against *E. coli* O157:H7 (5 mg/mL) and *S. aureus* (10 mg/mL). Its activity is linked to phenolic compounds and terpenes disrupting cell membranes, with efficacy varying based on bacterial cell wall differences (Panahi and Mohsenzadeh 2022). The antioxidant activity of FAEO‐loaded films is matrix‐dependent; chitosan films containing 3% FAEO showed a radical scavenging activity of 22% (Shokri et al. 2020), whereas the activity in methylcellulose‐based films was modulated by the nanoemulsion formulation. These observations underscore the need for systematic optimization of FAEO concentration and polymer matrix to achieve the desired balance of mechanical, barrier, and bioactive properties. The literature reveals a variation in the bioactivity of FAEO depending on the formulation (Moghaddam et al. 2018; Shokri et al. 2020), the antioxidant activity is consistently described as moderate to low (Ghasemi Pirbalouti et al. 2016; Shahbazi et al. 2016). This discrepancy can be explained by the chemical composition of FAEO, which is dominated by monoterpene hydrocarbons and contains low levels of phenolic compounds. Furthermore, the chemical composition of FAEO varies significantly depending on geographical origin, plant part, extraction method, and growing conditions (Ghasemi Pirbalouti et al. 2016; Shahbazi et al. 2016). For example, FAEO from the Kurdestan region showed higher antibacterial activity than samples from Lorestan (Shahbazi et al. 2016).

## 7 Applications of FAEO‐Loaded Packaging Materials

Packaging plays a crucial role in the food distribution chain by preserving food from contamination and microbial deterioration, minimizing food waste, and ensuring safety and hygiene (Abedi‐Firoozjah et al. 2025). Advancements in the packaging industry have resulted in the creation of food packaging solutions. Active packaging aids in preserving or augmenting the quality of processed and fresh products, hence extending their shelf‐life. Active food packaging incorporates functional components that interact with microbes and other biological systems, inhibiting the proliferation and functions of spoilage and pathogenic microorganisms (Abedi‐Firoozjah et al. 2025). According to studies on the FAEO, some chemical compounds and biological activity have been reported. The encapsulation of FAEO represents an effective strategy for its sustained release and improves its functional properties in food packaging. Several studies have shown that FAEO could function as an effective natural preservative in several food products. A study by Shokri et al. (2020) found that adding 3% FAEO into chitosan optimized Rainbow trout fillet preservation, with nanoemulsion coatings showing superior microbial and chemical stability (Shokri et al. 2020). Microbiologically, the total viable count (TVC) exceeded 7 log CFU/g in controls by day 8, but remained below this limit until day 12 in chitosan and EO‐coated samples, with nanoemulsions delaying spoilage further. The nanoemulsion coating reduced microbial growth by approximately 2.57 log CFU/g at day 16 (Figure 2). Chemically, TVB‐N levels, indicators of spoilage, stayed lower in FAEO‐treated samples, with nanoemulsions delaying the spoilage limit (25 mg N/100 g) until day 16, compared to day 8 in controls. Lipid oxidation measured via TBARS was also minimized in nanoemulsion‐coated fish, staying below 5 mg MDA/kg throughout storage, indicating enhanced antioxidant activity. Overall, FAEO nanoemulsion‐rich films extended shelf life of samples up to 16 days by effectively inhibiting microbial growth, lipid oxidation, and spoilage markers, demonstrating their potential to improve fish product preservation (Shokri et al. 2020).

**FIGURE 2:**
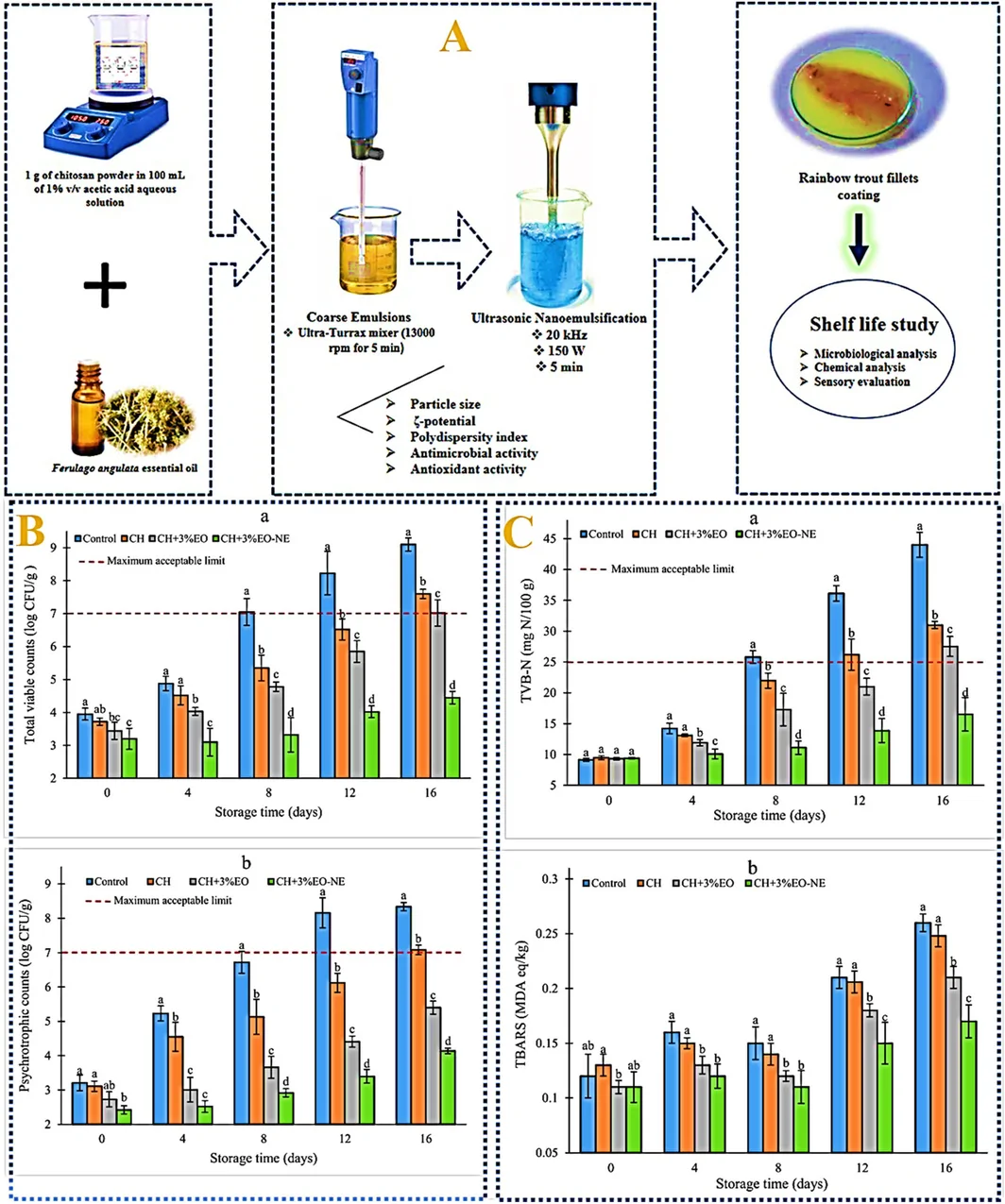
Schematic illustration of FAEO‐loaded chitosan coating (A). Effects of the proposed coating on total viable counts (Ba), psychrotrophic counts (Bb), TVB‐N value (Ca), and TBARS (Cb) value of fish samples during 16 days of storage at 4°C. Reproduced from Ref. Shokri et al. (2020) with permission.

In another study, Panahi and Mohsenzadeh (2022) demonstrated that incorporating FAEO into alginate coatings significantly extended chicken shelf life. In control samples, TVC exceeded 7 log CFU/g in controls by day 6 but remained below this limit until day 9 in FAEO‐treated samples (Panahi and Mohsenzadeh 2022). Psychrotrophic bacteria and Pseudomonas spp. increased more slowly in EO coatings, staying below spoilage levels until day 9–12. TBARS and peroxide value were notably lower in FAEO treatments, with TBARS reaching only 0.36 mg MDA/kg versus 1.20 in controls after 12 days. These results confirm that FAEO effectively inhibit microbial growth and lipid oxidation, significantly prolonging chicken freshness during storage for 12 days (Panahi and Mohsenzadeh 2022). Beigmohammadi et al. (2021) found that adding 0.5% FAEO to gelatin‐chitosan films effectively inhibited microbial growth in turkey meat, delaying spoilage. The initial TVC was 3.78 log CFU/g, reaching the 7 log CFU/g limit in controls by day 6, but remained below this threshold in treated samples up to day 12 (Beigmohammadi et al. 2021). Also, the treatment of samples using FAEO significantly reduced *S. aureus* and coliform counts, prolonging shelf life and improving sensory qualities. The antimicrobial effect is likely due to phenolic compounds like α‐pinene, making FAEO‐enhanced films a promising preservative for minimally processed foods. However, FAEO addition also influenced film color and transparency. This research group (2019) also developed starch films with 5%–15% CMC and 0.5%–1.5% FAEO. Reportedly, higher CMC improved tensile strength and barrier properties, while increased FAEO reduced strength but enhanced water resistance (Beigmohammadi et al. 2019). Films with higher CMC and FAEO slowed butter's peroxide value and acid number growth over storage, indicating extended shelf life of butter up to 6 days. These results suggest that starch‐CMC‐FAEO films effectively preserve butter, offering a sustainable alternative to aluminum foil. Mohammad Ganje et al. (2024) investigated the effects of chitosan and FAEO on mushroom shelf life. Chitosan coating significantly reduced tissue firmness loss, weight loss, and microbial growth, while FAEO intensified these effects. The combination of chitosan and 150 ppm FAEO showed the best results in preserving mushroom texture, color, and weight. This is attributed to the polysaccharide structure of chitosan, which blocks pores, reduces oxygen penetration, and inhibits cellular respiration and water loss. Further, the amino groups of chitosan have bacteriostatic effects, and the phenolic compounds in FAEO have antimicrobial activity (Ganje et al. 2024). Table 3 shows the physic‐mechanical properties and application of FAEO‐rich packaging films/nanofibers.

**TABLE 3:** The effect of FAEO on the physic‐mechanical and functional properties of biopolymer‐based packaging films.

Film base	FAEO content	Physic‐mechanical properties	Functional properties	Practical application		References
				Food model	Remarks	
Sodium alginate	10 and 20 mg/mL	NA	DPPH; IC50 value; 562 mg/mL MIC and MBC against *E. coli O157: H7, E. coli, S. aureus, B* *. cereus* , and *S. typhimurium*	Chicken breast	The lowest amount of TVB‐N, PV, TBARS, and microbial growth was related to FAEO‐rich alginate films	Panahi and Mohsenzadeh (2022)
Chitosan/gelatin	0.25% and 0.5%	Change Δ*E* (from 23.11 → 23.11 → 26.13), L⃰ (from 74.21 → 69.62), a (from −0.47 → 0.29), b (from 14.22 → 24.33)	Proper antibacterial activity against *S. aureus* and Coliform count	Turkey meat	Inhibiting the bacteria growth and enhancing the meat shelf‐life up to 15 days in cold condition	Beigmohammadi et al. (2021)
ZG/CMC	2.5 μL	Significantly reduced MC (from 11.64% → 8.00%–9.39%), WVP (from 2.108 × 10^−9^ → ~1.25 × 10^−9^ g.m/pa.h.m^2^), and EAB (from 12.55% → 6.10%). Increased TS (from 6.92 MPa → ~10.55–12.50 MPa) and YM (from 28.58 MPa → ~40–50 MPa)	NA	NA	NA	Razmjoo et al. (2022)
Zein	1% w/v	NA	NA	Butter oils	In wrapped sample with optimal films: lowest results for peroxide value 0.79–1.03 meq O_2_/Kg, TBARS 0.35–0.45 MDA/Kg, acid value 0.53–0.65 mg KOH/g	Shavisi (2024)
Chitosan	NA	NA	Potent antimicrobial ability against *S. aureus* and *E. coli* with MIC values of 0.45 and 2.12 mg/mL, respectively	NA	NA	Kaboudi et al. (2023)
Potato starch/CMC	0.5%, 1%, 1.5%	Solubility; 28.49% → 26.80%, MC; 14.10% → 13.79%, WVP; 1.62 × 10^−7^ g/m.h.pa → 1.49 × 10^−7^ g/m.h.pa, TS; 3.42 MPa → 3.37 MPa	NA	Butter	Reduction of acid number and peroxide number	Beigmohammadi et al. (2019)
Chitosan	0/5%	NA	NA	Mushroom	Reduced microorganisms and molds/yeasts; EO enhanced inhibition; quality maintenance	Ganje et al. (2024)
Chitosan	1%	NA	DPPH; up to 22% ± 0.19% Potent antibacterial activity against *Shewanella putrefaciens* and *Pseudomonas fluorescens*	Fish fillets	Inhibited bacterial growth, reduced TVB‐N and lipid peroxidation; improved texture, color, and overall acceptability	Shokri et al. (2020)

Abbreviations: CMC, carboxymethyl cellulose; EAB, elongation at break; IZ, inhibitory zone; MBC, minimum bactericidal concentration; MC, moisture content; MIC, minimum inhibitory concentration; NA, not analyzed; NS, not stated; TS, tensile strength; WVP, water vapor permeability; YM, Elastic modulus; ZG, Zedo gum.

Overall, the compiled data reveal that FAEO incorporation extends the shelf life of perishable foods by an average of 3 to 8 days across all food matrices studied, with the highest efficacy observed in fish and poultry products. Most studies that conducted statistical analyses reported significant improvements (*p < 0.05*) in microbial and chemical quality parameters.

## 8 Release Kinetics and Diffusion Mechanisms

The migration of a functional agent from packaging systems into food arises from mass transfer phenomena, combining equilibrium and kinetic processes across the food, headspace, and polymer matrix. The release kinetic occurs through two primary mechanisms. The release rate of an active agent from a polymer film must align with food spoilage kinetics. Too slow release provides insufficient protection, while too fast release wastes the agent through degradation. Therefore, diffusion must be tailored to the specific requirements of each food product (Rojas et al. 2023).

Spray‐dried chitosan microspheres loaded with FAEO exhibited a biphasic release pattern (Bahrampour et al. 2023). This pattern indicates an initial burst release followed by a sustained release phase, which is advantageous for active packaging requiring immediate antimicrobial action followed by long‐term protection. The microspheres were highly soluble and hygroscopic. FAEO was physically entrapped within chitosan microparticles via hydrogen bonding and hydrophobic interactions, as confirmed by FTIR. Release followed a biphasic pattern: an initial fast phase (first 5 h, ~50% EO released) due to swelling of non‐cross‐linked chitosan, followed by slower release via surface erosion and diffusion. Higher molecular weight chitosan created denser particle shells, significantly reducing the release rate compared to low molecular weight chitosan. In another study, chitosan nanoparticles loaded with FAEO prepared by ionic gelation technique demonstrated successful physical entrapment of FAEO (Kaboudi et al. 2023). Using ionic gelation technique with a chitosan to FAEO ratio of 1:1.25, a maximum encapsulation efficiency of 60.20% and loading capacity of 24.5% were achieved. Thermogravimetric analysis (TGA) showed that encapsulated EO decomposed at a higher temperature than its free form, indicating successful stabilization of FAEO within chitosan nanoparticles. FAEO interacts physically with chitosan, as confirmed by FTIR peaks at 3310 cm^−1^ (C‐H stretching of aldehydes) and 890 cm^−1^ (pyranose ring). Here, FAEO from nanoparticles showed initial rapid release (within 30 min) from surface layers, followed by steady diffusion from the polymer matrix up to 180 min. Lower pH (at 3) enhanced chitosan degradation, giving 36% total release, compared to 27% at pH 7.4. Release reached a plateau after 250 min. Initial burst release from surface‐bound EO, followed by sustained diffusion‐controlled release from the chitosan matrix, with faster release at acidic pH due to polymer degradation (Kaboudi et al. 2023). Shavisi (2024) found encapsulation efficiencies of FAEO in zein nanofibers ranging from 96.15% to 96.82%. Release was temperature‐dependent; higher temperatures increased macromolecular chain mobility, accelerating release. A biphasic pattern was observed: initial rapid release (13.23%–23.45% within 12 h) due to solution‐induced matrix disruption, followed by slower release (31.54%–39.95% at 48 h) attributed to the dense nanofiber structure (Shavisi 2024).

The choice of polymer matrix also profoundly influences the release kinetics of FAEO. For instance, while spray‐dried chitosan microspheres exhibited a biphasic release pattern with an initial burst followed by a sustained phase (Bahrampour et al. 2023), the release from chitosan nanoparticles prepared by ionic gelation was highly pH‐dependent, with faster release at acidic pH due to polymer degradation (Kaboudi et al. 2023). In contrast, encapsulation in zein nanofibers via electrospinning resulted in a temperature‐dependent release, where higher temperatures accelerated release due to increased macromolecular chain mobility (Shavisi 2024). These findings collectively indicate that the release mechanism, whether diffusion‐controlled, swelling‐controlled, or degradation‐controlled, is dictated by the polymer's physicochemical properties, necessitating tailored approaches for different food applications.

On the other hand, the encapsulation technique plays a pivotal role in determining the efficacy of FAEO‐loaded packaging systems. Electrospinning, for instance, offers a promising approach to fabricate nanofibrous structures capable of encapsulating bioactive compounds with high efficiency (Shavisi 2024). Shavisi (2024) demonstrated encapsulation efficiencies of FAEO in zein nanofibers ranging from 96.15% to 96.82%, showcasing the high loading capacity of this technique (Shavisi 2024). In comparison, the ionic gelation technique for chitosan nanoparticles achieved a maximum encapsulation efficiency of 60.20% and a loading capacity of 24.5%. While electrospinning offers superior encapsulation efficiency, the ionic gelation method provides benefits such as simplicity and mild processing conditions (Kaboudi et al. 2023). Spray‐drying, another widely used technique, resulted in stable chitosan microspheres with encapsulation efficiency exceeding 70% and uniform particle size (Bahrampour et al. 2023). The choice of method thus involves a trade‐off between encapsulation efficiency, scalability, and the desired release profile.

## 9 Comparison of FAEO With Other Functional Additives

In the field of active packaging systems, EO are widely used as natural antimicrobial/antioxidant agents (Wang et al. 2023; Zhang et al. 2025). These molecules exhibit potent bactericidal activity against a broad spectrum of foodborne pathogens and potent antioxidant properties, making them highly effective for preserving fatty foods (Chen et al. 2023; Friedman et al. 2002). Compared to thymol, other EO components such as eugenol, carvacrol, and cinnamic acid exhibit lower antibacterial potency (Sivaram et al. 2022). In contrast, FAEO is dominated by monoterpene hydrocarbons like α‐pinene and Z‐β‐ocimene, which provide moderate antimicrobial effects (Shavisi 2024; Shokri et al. 2020). Yu et al. (2023) developed zein films with cinnamaldehyde, carvacrol, or eugenol for active packaging. EOs improved mechanical properties better than oleic acid, though with weaker water barrier. Cinnamaldehyde films showed strongest antimicrobial activity against *S. aureus* (inhibition zones up to 16.67 mm). The films released EO gradually over 96 h (Yu et al. 2023). In another work, treating vacuum‐packed anchovy fillets with thymol or eugenol extended their edible shelf life from 8 days (control) to 19 days, while carvacrol and α‐terpineol achieved 15 days. The treated fish fillets showed significantly lower lipid oxidation, fewer biogenic amines, and better sensory scores, preserving the natural quality of the anchovy during 22 days of refrigerated storage (Ozogul et al. 2024). Regarding antioxidant activity, clove (Chen et al. 2023), cinnamon (Chen et al. 2024), and eugenol (Huang et al. 2022) oils demonstrate high free radical scavenging capacity due to their phenolic hydroxyl groups. FAEO, however, shows lower antioxidant activity (Shokri et al. 2020). Despite these differences, FAEO offers distinct advantages for certain packaging applications. Its milder antimicrobial action and lower phenolic content result in less sensory impact on packaged foods. A study demonstrated that chitosan‐gelatin films containing 0.5% FAEO significantly extended the shelf life of turkey fillets while actually improving sensory properties (Beigmohammadi et al. 2021). This contrasts with clove (Javed et al. 2025) or thyme (Boskovic et al. 2019) and apricot kernel (Priyadarshi et al. 2018) oils, FAEO exhibited weaker synergy with chitosan against microbes. Other studies have similarly reported its moderate antimicrobial activity. This likely stems from its lower levels of phenolic compounds, such as carvacrol and thymol, which are key contributors to antimicrobial effects in Eos (Shokri et al. 2020). However, as a significant note, recent reviews indicate that EO release from carrier matrices is governed by matrix structure. Rapid release causes early potency loss and strong odor, while excessively slow release provides inadequate protection against spoilage. Thus, properly designing carrier systems to tune release profiles is crucial for achieving both antimicrobial effectiveness and sensory tolerance (Godini et al. 2026).

## 10 Challenges and Safety Aspects

The integration of FAEO into food packaging systems offers promising benefits, but several significant challenges must be addressed to enable their widespread adoption and commercial viability. A primary limitation of FAEO, is its high volatility and susceptibility to degradation under environmental stresses such as light, heat, and oxygen. This instability can lead to rapid loss of bioactive compounds, diminishing functional activity during storage and application. Furthermore, the strong aroma and flavor profile of FAEO, while beneficial at low concentrations, can become overpowering or undesirable at higher dosages, potentially affecting the sensory qualities of packaged foods (Borah et al. 2024; Sardar et al. 2024). FAEO is inherently hydrophobic, resulting in poor water solubility and limited compatibility with hydrophilic biopolymer matrices commonly used in sustainable packaging (e.g., starch, cellulose, chitosan). This incompatibility can cause uneven distribution, phase separation, or migration of the oil within the film, leading to inconsistent functional properties, reduced mechanical strength, and compromised barrier performance (Borah et al. 2024; Sardar et al. 2024). Achieving homogeneous incorporation and stable dispersion of FAEO in packaging materials remains a technical challenge. While low concentrations of FAEO can enhance sensory attributes, higher levels may impart strong or atypical flavors and aromas that are not well accepted by consumers. This is particularly critical in foods with subtle flavor profiles or where the EO's herbal notes may clash with the product's intended taste. Sensory optimization is thus essential, requiring careful balance between functional efficacy and consumer preferences (Sardar et al. 2024). The use of FAEO in food packaging must comply with food safety regulations, which vary across regions and countries. Comprehensive toxicological assessments and migration studies are required to ensure that the EO and its constituents do not pose health risks when in contact with food. Regulatory approval processes can be lengthy and costly, potentially delaying commercialization (Sardar et al. 2024). Although laboratory‐scale studies demonstrate the potential of FAEO‐loaded films, translating these findings to industrial‐scale production presents economic and technical hurdles. Extraction of high‐quality FAEO in sufficient quantities, standardization of chemical composition, and the development of scalable encapsulation or emulsion technologies are all needed to ensure cost‐effective manufacturing (Borah et al. 2024). Lack of standardized protocols for extraction, characterization, and application further complicates comparative studies and regulatory approval (De Farias et al. 2025). According to the European Framework Regulation (EC) No. 1935/2004, food contact materials must not endanger human health or alter food composition (Baiguini et al. 2011). For plastic materials, Regulation (EU) No. 10/2011 establishes an overall migration limit (OML) of 60 mg/kg food or 10 mg/dm^2^ surface area for the total sum of all migrating substances (EFSA Panel on Food Contact Materials, Enzymes and Processing Aids (CEP), et al. 2020). Karimian et al. (2014) assessed the cytotoxicity of *F. angulata* leaves hexane extract on MCF‐7 human breast adenocarcinoma cells using MTT assay, reporting an IC50 value of 5.3 ± 0.82 μg/mL, while acute toxicity studies confirmed the safety of the plant extract (Karimian et al. 2014). Hajimohammadi et al. (2024) administered oral doses of 100, 200, and 400 mg/kg to rats for 10 days, with toxicological evaluation showing no side effects in liver, lung, heart, and kidney tissues (Hajimohammadi et al. 2024). Ekin et al. (2019) investigated the nephroprotective effect of flower extract at 150 and 300 mg/kg in a Dimethylnitrosamine‐induced rat model, reporting no observed toxicity at 300 mg/kg (Ekin et al. 2019).

## 11 Industrial Aspects and Future Prospects

Based on our findings and the current state of the literature, the following perspectives are proposed for future research and industrial development. Industrial‐scale encapsulation via spray‐drying achieves yields above 50% and efficiencies exceeding 70% (Bahrampour et al. 2023).

Additional studies should investigate the effectiveness of FAEO‐loaded active materials against foodborne viruses such as norovirus or rotavirus, as well as against parasites like Cryptosporidium or Giardia. Most studies on FAEO‐based packaging films have used non‐pathogenic microbes (e.g., *S. aureus* or non‐toxigenic *E. coli* ). Future research must distinguish between preventing general spoilage versus inhibiting true foodborne pathogens. Additionally, microbial shelf‐life extension should be separated from biochemical degradation effects during storage. Proposed solutions to address scale‐up challenges are: (i) encapsulation‐based extrusion, (iii) droplet coalescence control, and (iii) compatibility optimization between FAEO and polymer matrices. These approaches aim to overcome barriers in mass production, reproducibility, and long‐term stability. Future research should also pursue synergistic FAEO‐bioactive blends for improved antimicrobial and antioxidant activity. Further, novel approaches should be identified to reduce FAEO loss from films due to evaporation in food packaging applications, thereby extending its release into the atmosphere surrounding packaged foods.

## 12 Conclusion

This review demonstrates that FAEO possesses promising antimicrobial and moderate antioxidant properties, which support its potential as a natural additive for active food packaging systems. Incorporation of FAEO into biopolymer‐based films and nanostructures has been shown to enhance shelf life and improve food safety in products such as fish, poultry, dairy, and mushrooms. This review pinpoints key performance factors such as polymer matrix, droplet dispersion, packaging methods, encapsulation technique, environmental conditions, and nanoparticle stabilization, uncovering design principles for optimized packaging. It also identifies research gaps and suggests practical future directions. However, several critical limitations must be addressed before commercial application. These include high volatility, poor water solubility, potential sensory impacts, and lack of regulatory approvals. Further, the chemical composition and bioactivity of FAEO vary considerably depending on geographical origin, extraction methods, and plant parts, which pose challenges for standardization. Future studies must establish standardized protocols for antimicrobial, antioxidant, and sensory evaluation. To achieve this, advanced encapsulation ensures uniform FAEO dispersion and controlled release. Further, FAEO loading must be optimized for high‐moisture and fatty foods. However, only scarce data are available regarding the application of FAEO in real food systems, which makes comparative performance assessment somewhat challenging. Suggested methods to solve industrial scaling obstacles include extrusion combined with encapsulation and enhancing FAEO‐biopolymer compatibility. These approaches target present hurdles in mass manufacturing, consistent functionality, and extended storage durability. Future studies should investigate hybrid active packaging systems that blend FAEO with other bioactives, harnessing synergy to boost both antimicrobial and antioxidant efficacy. Several critical research priorities demand focused investigation to advance FAEO‐based active packaging. Systematic electrospinning studies across diverse polymer matrices are needed to identify optimal carriers for controlled release. Industrial scalability must be advanced through extrusion‐based processing and three‐dimensional (3D) printing technologies, which offer promising routes for continuous manufacturing of FAEO‐loaded packaging materials. More so, the evaluation of FAEO‐based packaging should extend beyond fish, poultry, and dairy to include fresh fruits and vegetables, where post‐harvest preservation challenges differ significantly and remain largely unexplored. Synergistic combinations with nanomaterials and other bioactives warrant systematic investigation to enhance antimicrobial and antioxidant performance through complementary mechanisms. Artificial intelligence (AI) and machine learning approaches present promising opportunities for predicting optimal FAEO compositions, modeling release kinetics, and designing customized packaging architectures based on specific food requirements. Finally, comprehensive safety assessments, including migration studies and long‐term stability evaluations, are essential prerequisites for regulatory approval and commercial adoption. Therefore, while FAEO is a promising alternative to synthetic preservatives, future research should focus on: (i) optimizing encapsulation techniques to improve stability and controlled release; (ii) standardizing extraction and characterization protocols; (iii) conducting comprehensive toxicological and migration studies for regulatory approval; and (iv) evaluating cost‐effectiveness for industrial‐scale production. Only through such balanced and rigorous investigation can FAEO‐based packaging materials transition from laboratory research to practical, sustainable food packaging solutions.

## Author Contributions

**Mahshid Bahraminejad:** investigation, writing – original draft, writing – review and editing. **Delara Moradi Mirhesari:** investigation, writing – original draft, writing – review and editing. **Ehsan Sadeghi:** writing – original draft, writing – review and editing, investigation. **Behnam Bahramian:** writing – original draft, investigation, writing – review and editing. **Reza Abedi‐Firoozjah:** investigation, writing – original draft, writing – review and editing, conceptualization, methodology. **Samira Shokri:** conceptualization, investigation, writing – original draft, writing – review and editing, validation, methodology, project administration.

## Funding

The authors have nothing to report.

## Conflicts of Interest

The authors declare no conflicts of interest.

## Data Availability

No new data were generated or analyzed in this review article. All data referenced in this paper are from previously published studies, which are cited in the reference list.
